# DNA replication: Mechanisms and therapeutic interventions for diseases

**DOI:** 10.1002/mco2.210

**Published:** 2023-02-05

**Authors:** Hao‐Yun Song, Rong Shen, Hamid Mahasin, Ya‐Nan Guo, De‐Gui Wang

**Affiliations:** ^1^ School of Basic Medical Sciences Lanzhou University Lanzhou Gansu China

**Keywords:** DNA damage response, cancer, DNA replication, posttranslational modifications (PTMs), replication stress

## Abstract

Accurate and integral cellular DNA replication is modulated by multiple replication‐associated proteins, which is fundamental to preserve genome stability. Furthermore, replication proteins cooperate with multiple DNA damage factors to deal with replication stress through mechanisms beyond their role in replication. Cancer cells with chronic replication stress exhibit aberrant DNA replication and DNA damage response, providing an exploitable therapeutic target in tumors. Numerous evidence has indicated that posttranslational modifications (PTMs) of replication proteins present distinct functions in DNA replication and respond to replication stress. In addition, abundant replication proteins are involved in tumorigenesis and development, which act as diagnostic and prognostic biomarkers in some tumors, implying these proteins act as therapeutic targets in clinical. Replication‐target cancer therapy emerges as the times require. In this context, we outline the current investigation of the DNA replication mechanism, and simultaneously enumerate the aberrant expression of replication proteins as hallmark for various diseases, revealing their therapeutic potential for target therapy. Meanwhile, we also discuss current observations that the novel PTM of replication proteins in response to replication stress, which seems to be a promising strategy to eliminate diseases.

## Graphical abstract

Accurate DNA replication is modulated by multiple replication‐associated proteins, which is fundamental to preserve genome stability. Abundant replication proteins are involved in tumorigenesis and development, implying these proteins act as therapeutic targets in clinical. Replication‐target cancer therapy emerges as the times require. Furthermore, the novel posttranslational modification of replication proteins in response to replication stress, which seems to be a promising strategy to eliminate diseases.

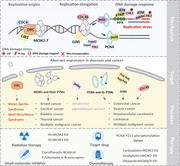

## 1 INTRODUCTION

Accurate, faithful, and error‐free DNA replication is a vital prerequisite to ensure normal operation for the entire biological processes. DNA replication is an intricate and ingenious procedure that is fundamental to cellular life. Incomplete or erroneous DNA replication events lead to aberrated cell cycles, gene mutations, and gene copy number variations, further resulting in diseases, even cancer.^1^, ^2^

DNA replication can be roughly separated into three typical sections: (1) DNA replication initiation, in which the replication origins are prepared to unwind the DNA helix; (2) DNA replication elongation, in which replisomes move in opposite directions via semi‐conservative synthesis; (3) DNA replication termination, when converging replication forks meet and replisome disassembly.^3^, ^4^ Integrated DNA replication events are tightly regulated from bacteria to eukaryotic cells to allow correct genetic information transmission through cell division. In whole process of DNA replication, random mistakes are a source of genomic instability, causing heritable mutations that drive cancer evolution.^5^

Owing to aberrant DNA replication and constitutive growth signaling, cancer cells may experience “replication stress,” a phenomenon that delays DNA synthesis and is a hallmark of cancer.^6^, ^7^ To safeguard precise duplication of the entire genome, cells initiate the DNA damage response (DDR) mechanisms to account for the continuous barriers.^8^ The DNA repair pathways in mammalian cells accurately repair distinct types of DNA damage, whereas DNA repair dysfunction can predispose organisms to disease. Nevertheless, the DDR system may be defeated to maintain the genomic integrity due to oncogenes activation or tumor suppressor genes inactivation. Therefore, exacerbating DNA replication stress (RS) as well as targeting DNA repair defects in cancer cells is an effective strategy for treating cancer specifically.^9^

Posttranslational modifications (PTMs) of proteins could affect their functions in positively or negatively way, impacting multiple biological processes such as DNA replication, gene transcription, and DDR.^10^, ^11^ Recent studies supported that PTMs of replication factors have an extraordinary effect on DNA replication and respond to RS. Thus, the advanced understanding of modification of replication licensing factors and their implications for DDR may provide a novel insight into the cancer therapeutic target.

In this review, we elaborate on the overall DNA replication mechanism and summarize the comprehensive approaches that are aiming harness RS to target cancer. Furthermore, we explore the latest strategies and novel ideas to improve the efficacy and specificity of anticancer therapies. Meanwhile, we also enumerate the multifarious PTMs, elaborating how PTMs of replication proteins mediated DNA replication, RS response, DNA damage repair, and oncogenesis mechanism, which may provide a polynary insight into tumorigenesis and tumor therapeutics.

## 2 THE BASICS OF EUKARYOTIC DNA REPLICATION

### 2.1 DNA replication initiation

Mini‐chromosome maintenance (MCM) proteins are composed of six subtypes, MCM2, MCM3, MCM4, MCM5, MCM6, and MCM7. All subunits integrate into a hetero–hexameric complex and act as a replicative DNA helicase to unwind the parental DNA.^12^ Beyond that, other MCM proteins, MCM8, MCM9, and MCM10, are reportedly essential for the DNA replication and remaining genome maintenance.^13^, ^14^, ^15^

**FIGURE 1:**
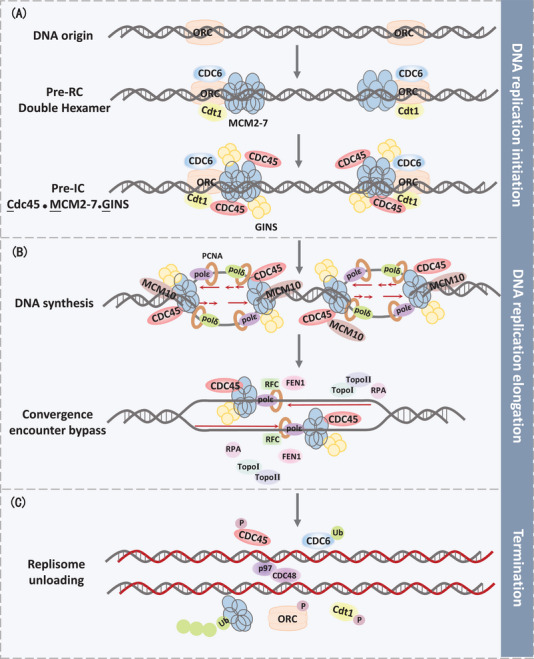
The schematic diagram for DNA replication. (A) DNA replication initiation procedures are described in the text. In the early G1 phase, cell division cycle 6 (CDC6) and DNA replication factor 1 (Cdt1) are recruited to replication origins, subsequently cooperating with MCM2‐7 to load onto chromatin. MCM2‐7 complex interacts with GINS and Cdc45 as CMG complex to initiate DNA replication. (B) DNA replication elongation. CMG helicase initiates double‐strand DNA unwinding. Two replicative polymerases, polε and polδ, rely on PCNA to principally execute DNA synthesis to elongate the nascent leading and lagging strands, respectively. Additionally, flap endonuclease 1 (FEN1) and topoisomerases (TopoI, TopoII) safeguard typical and efficient DNA polymerization. (C) DNA replication termination. CMG removal from the strand represents replication termination. CDC48/p97‐mediated MCM7 polyubiquitination and CDK‐mediated ORC phosphorylation facilitate CMG disassembly, thus leading to CMG unloading.

In eukaryotic cells, activation of replication origins is a prerequisite of DNA replication, manifesting the bidirectional movement.^16^, ^17^ The prereplication complex (pre‐RC) forms at the origin recognition complex (ORC1‐6), which serves as the actuated operator of DNA replication. In order to maintain genome integrity, ORC proteins are essential for establishing pre‐RC at origins since the distribution and density of origins have to be adequate to replicate the entire genome without leaving any regions un‐replicated. In the early G1 phase, cell division cycle 6 (CDC6) and DNA replication factor 1 (Cdt1) are recruited to the replication origins, subsequently attracting MCM2‐7 complex to load onto chromatin.^18^, ^19^ MCM2‐7 hexamer itself has restricted helicase activity, while executing integrated helicase activity in combination with Cdc45 and GINS (CMG) during G1/S transition.^20^, ^21^ Those proteins could compose of preinitiation complex (pre‐IC), which then preparing to form bi‐directional replication forks once MCM double hexamers separating into two single units.^22^ Additional factor, MCM10, collaborates with polymerase ε (polε) and polymerase δ (polδ) in replication origins for replication initiation, meanwhile interacting with CMG helicase to stabilize the replisome.^23^, ^24^ Moreover, one recent study found that MCM10 is necessary for CMG to transit between double‐strand DNA (dsDNA) and single‐strand DNA (ssDNA). Additionally, MCM10 migrates along with the replication fork and energizes replication elongation (Figure 1A).

In light of the bulky size of genomes and abundant amounts of chromosomes, eukaryotic cells contain numerous replication origins to duplicate their genomes. Nonetheless, massive replication origins assist high‐efficiency genetic information transfer with more hazard since their distribution and proceeding have to be tightly controlled. Eukaryotic cells have an extensive system to guarantee precise DNA replication. During the S phase, disparate genome regions or domains are duplicated at a staggered time, and the origin licensing system is carried out from firing at distinct cell cycle phases. In addition, in the G1 phase, license origins are much more than they used in the subsequent S phase, while the inactive origins are named as “dormant origins.” The plain fact is that dormant origins constitute the tremendous majority of licensed origins, which serve as backup to sustain the replication fork regular progression under conditions of RS.

Since overabundance and distribution patterns on chromatin, the definite and accessional functions of MCM proteins are always contradictory. The issue is defined as “MCM paradox,” which is chiefly embodied in two aspects: (1) MCM2‐7 complexes massively exist in nonreplicated DNA; (2) excess MCM hetero–hexamers attach to chromatin instead of replication origins and ORCs.^25^, ^26^ Apparently, the excess MCMs are involved in other biological processes. Numerous studies proved that MCMs serve as biomarkers in multiple tumors, which are closely related to tumorigenesis, development, and even in tumor therapeutic.

### 2.2 DNA replication elongation

Afterward, dozens of distinct proteins consistently coordinate to promote DNA replication elongation. Owing to the DNA antiparallel structure and DNA polymerases’ 3′–5′ direction of forward motion, the running replication forks separate into two single strands, which are continuously synthesized leading strand and inconsecutive synthesized lagging strand, respectively.^27^ In the lagging strand, discontinuous and short fragments are considered as Okazaki fragments, which require DNA ligase to assemble into the complete lagging strand rapidly and ultimately.^28^

DNA elongation and polymerization is catalyzed by multifarious enzymes, which are responsible for DNA synthesis and progression of the DNA replication. Polymerase α (polα)/primase mainly partakes in the initial stage of DNA synthesis.^29^ Four subunit enzymes of polα/primase catalyze RNA oligonucleotide synthesis, which subsequently can be applied to extend by a short stretch of DNA. After this initiation step, polα is immediately switched into replicative polymerase via an ATP‐dependent manner.^30^ Two replicative polymerases, polε and polδ, principally execute DNA synthesis to elongate the nascent leading strand and lagging strand, respectively. Both polε and polδ are four subunit enzymes with intrinsic 3′–5′exonuclease proofreading activities, which increase replication fidelity with a lower mutation rate.^31^, ^32^ Moreover, multiple evidence suggested that polymerase activities of polδ are stimulated by protein proliferating cell nuclear antigen (PCNA), serving as a platform to coordinate numerous proteins interaction at the replication fork. Polδ cooperates with PCNA to promote long stretches of DNA synthesis.^33^ Nevertheless, PNCA could not load onto DNA without replication factor C (RFC) assistance, which could wrap PCNA homo‐trimeric ring to promote its DNA loading via ATP‐dependent manner.^34^ Additionally, flap endonuclease1 (FEN1) and Dna2, two endonucleases, are mainly needed for DNA and RNA flap structure cleaving, which are mediated by replication protein A (RPA).^35^ Ultimately, flap cleavage generated DNA nick is sealed by DNA ligase I (Figure 1B).^36^

### 2.3 DNA replication termination

In contrast to the initiation and elongation steps, DNA replication termination still remains several queries, even though it occurs on neighboring replication origins encounter. Due to the torsional strain caused by DNA helicase, positive supercoils structure must be removed by DNA topoisomerases to maintain the replication fork progression and genomic integrity. Type I and type II topoisomerases unwrap supercoils primarily to rotate the direction of fork evolution into clockwise, which could transfer the topological stress. Additionally, type II topoisomerase specifically removes precatenanes to assure converging replisomes unwind and DNA complete synthesis.^37^, ^38^

Like the ultraprecise instrument, every module of DNA replication all links with one another. During the S phase, reloading of MCMs is inhibited to ensure that no genome segment is re‐replicated to preserve genome integrity.^39^ Except for MCM proteins, several proteins are involved in this node. Cdt1, as the component of pre‐RC, is the prime modulator to prevent re‐replication.^40^ CDK‐mediated phosphorylation of Cdt1 is inhibited from interacting with Orc6 once the DNA replication initiation in *Saccharomyces cerevisiae*.^41^ In eukaryotes, however, Cdt1 is degraded upon S phase entry through two independent ubiquitin‐mediated pathways.^42^, ^43^ In addition, other components of pre‐RC, ORC1‐6, are also the critical point for preventing re‐replication.^44^ During S phase entry, ORC1 is released from replicating sequence via CDK‐mediated manner, which prevents ORC from entering second round of licensing.^45^ CDC6 also manifests in preventing re‐replication via a distinct mechanism. Some studies support that the SCF^cyclin F^ ubiquitin ligase complex impedes DNA re‐replication by proteasomal degradation of CDC6 in the cell cycle.^46^

In eukaryotes, neighboring CMG complexes meet each other on different strands, which is propitious to stable and orderly replication progression. Leading and lagging strand separation promotes CMG of one replisome to straightway transfer into the lagging strand template. Rapid encounter of adjacent CMG complex without pausing could preserve genome stability, whereas suspending for a while when CMG complex confronts a covalent DNA–protein.^47^ This observation points out that cells set the defense mechanism to prevent conflict between adjacent CMG complexes during DNA replication termination.

CMG removal from the strand is a remarkable event in eukaryotic replication termination when CMG cooperated with dsDNA. Previous reports suggested that converging CMG complexes proceed migration along the leading strand template until the downstream Okazaki fragment, which no longer performs dsDNA unwinding at all. Ultimately, CRL2^Lrr1^‐mediated MCM7 polyubiquitination leads to CMG unloading, subsequently removed by CDC48/p97 segregase (Figure 1C).^48^

DNA replication is an intricate process with a coordinated interplay of multiple proteins. As we summarized, each step of DNA replication must be strictly regulated to preserve genome integrity, while internal or external DNA‐damage agent always threatens DNA replication to activate DDR system. Meanwhile, dysfunction of DNA replication and DDR causes severe diseases, which highlights the role of DNA replication in tumorigenesis and development.

## 3 EVOLUTION OF THE CORE REPLICATION PROTEINS

### 3.1 CMG complex

**FIGURE 2:**
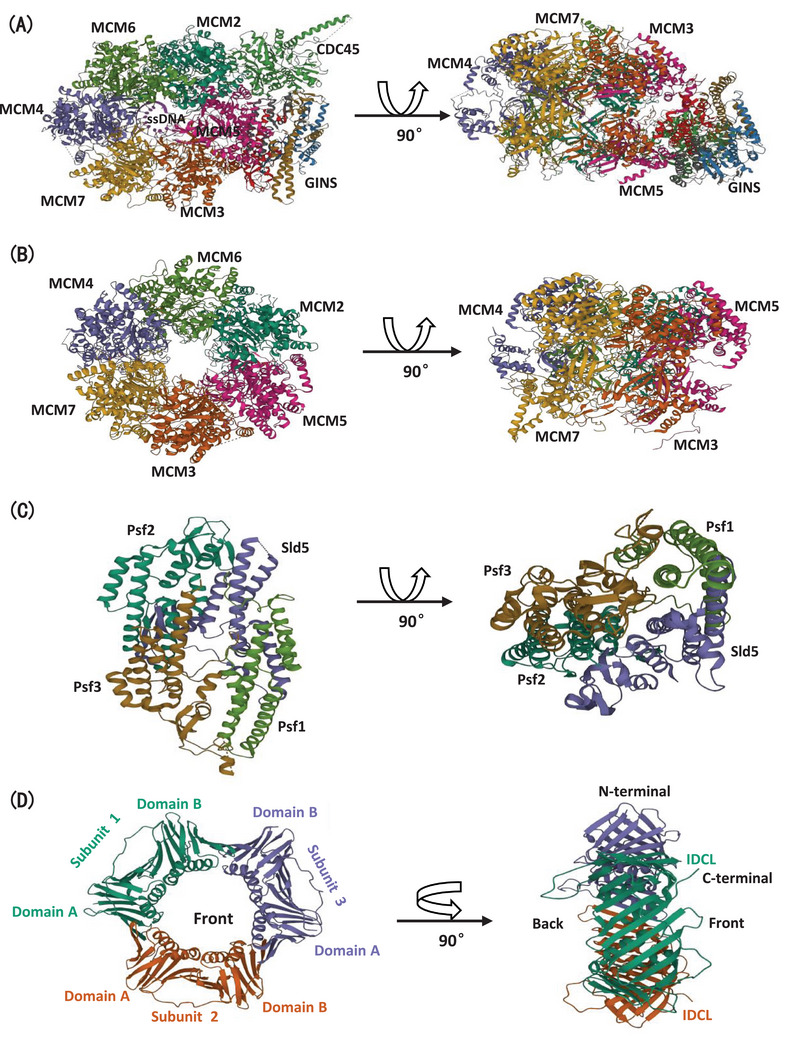
General crystal structure of CMG complex and PCNA. (A) Crystal structure of the CMG complex. The single‐strand DNA (ssDNA) is colored lavender and each CMG units are uniquely colored and labeled. (Pictures from Protein Data Base mark as 6XTX. 3D PFV: 6XTX (rcsb.org).) (B) Crystal structure of the MCM2‐7 complex. MCM units are uniquely colored and labeled. (Pictures from Protein Data Base marks as 3J48. RCSB PDB ‐ 3JA8: Cryo‐EM structure of the MCM2‐7 double hexamer.) (C) Crystal structure of GINS. GINS units are uniquely colored and labeled. (Pictures from Protein Data Base mark as 2Q9Q.RCSB PDB ‐ 2Q9Q: The crystal structure of full‐length human GINS complex.) (D) Crystal structure of PCNA as viewed from top and side. Three subunits are uniquely colored and labeled. (Pictures from Protein Data Base mark as 3JA9. RCSB PDB ‐ 3JA9: Structure of native human PCNA.)

In eukaryotes, DNA replicative helicase CMG complex binds to dsDNA at replication origins, subsequently transfers to ssDNA for DNA unwinding. As we described above, Cdc45 and GINS cooperate with MCM2‐7 during S phase entry, forming CMG helicase for bidirectional replication forks (Figure 2A).^49^

MCM proteins were firstly identified in *S. cerevisiae*, which were deemed to MCM. Based on electron microscopy investigations, each MCM monomer involves two conserved main domains exercising respective functions.^50^, ^51^ MCM2‐7 complexes could motion via the nuclear localization signals on N‐terminal region of MCM2 and the C‐terminal of MCM3, whereas nuclear export signals on the central part of MCM3.^52^ In contrast, recent data suggest that MCM complexes are shuttled in interphase cells basically relying on the nuclear export signals in MCM3.^53^

The N‐terminal domain (MCM_N_) possesses three consistent crystal structure subdomains: an OB (oligonucleotide/oligosaccharide binding)‐fold, a peripheral helical bundle, and usually a zinc‐binding motif (Figure 2B).^54^, ^55^

The OB‐fold subdomain links two single hexamers as head‐to‐head form and is also for DNA binding.^51^ Mcm4^Chao3^ (chromosome aberrations occurring spontaneously 3) mutation occurring in mouse Mcm4 OB fraction disrupts routine DNA binding process, resulting in genomic instability.^56^, ^57^

The peripheral helical bundle interacts with the OB fraction via a slight linker promoting the interaction with DNA.^58^ The observation indicated that a helical bundle might be essential for protein–protein interplay and protein–DNA interactions during the initiation step. However, molecular mechanics studies presented that deleting a helical bundle exerts a limited influence on MCM function.^59^, ^60^

The X‐ray crystal structures of MCM_N_ suggested a zinc‐binding domain, which presented two conserved arginine residues in *Pyrococcus furiosus* MCM (*pf*MCM). Studies with the *pf*MCM verified that the zinc‐binding domain is probably needed for ssDNA binding.^54^ Mutation of these two conserved arginine residues in MCM4/6//7 interfered with the loading of MCM2‐7 complex onto DNA, further resulting in growth defect in *S. cerevisiae*. These findings suggested that zinc‐binding domain of MCM4/6/7 is the vital region in ssDNA binding and origin melting.^61^, ^62^ In eukaryotes, the zinc‐binding motif of MCM3 lacking a prominent motif impacts the MCM2‐7 complex original function, suggesting that zinc‐binding motifs play a vital role in MCM2‐7 activities.^63^

MCM proteins contain an AAA^+^ ATPase domain in C‐terminal with two subunits terming as Walker A and Walker B, which are integrant for ATP hydrolysis and ATP binding.^64^, ^65^ Mutation of nearly any residues of the MCM AAA^+^ ATPase domain eradicates ATPase activity.^66^ Despite all the MCMs harboring ATP‐binding motifs at the intersubunit interfaces, the ATP‐binding mode is quite different.^67^ MCM4/6/7 proteins exhibit distinct functions when the ATP binding sites undergo mutations.^68^ It should also be mentioned that MCM7 is required for ATP hydrolysis and DNA helicase via its ATP binding motif.^69^

Nuclear magnetic resonance (NMR) structure studies revealed that the C‐terminus of MCMs comprises a winged helix (WH) domain. Furthermore, the WH domain connecting to the AAA^+^ ATPase domain exhibits ATPase activity, promoting the domain shift via a flexible linker with the protein core.^70^, ^71^ In contrast, archaeal MCM exhibits increasing ATPase activity and dsDNA unwinding activity when partial deleting of WH domain.^72^ Thus, WH domain may reserve the latent function during dsDNA unwinding and may take effect in initiating helicase activity.^73^

Except for conserved MCM2 and MCM3, MCM8 and MCM9 also possess a nuclear localization signal to shuttle between cytoplasm and nuclear.^74^ Some studies indicated that MCM2 and MCM3 distribute in the cytoplasm but temporally and spatially shift to nucleus in a cell cycle‐dependent manner.^52^ However, the distribution of MCM2 may also be associated with DNA damage. Envelope protein gp70 directly recognized MCM2 nuclear localization signal in the cytoplasm, thus enhancing DNA damage‐induced apoptosis.^75^, ^76^ However, limited researches discuss the purpose of the MCM proteins motion.

The eukaryotic GINS complex consists of four subunits, Sld5, Psf1, Psf2, and Psf3, pronounced as Sld‐go, Psf‐ichi, Psf‐ni, and Psf‐san in Japanese. Despite its central role in CMG complex, GINS also modulates massive protein interaction during DNA replication and DNA repair.^77^ Each subunit of GINS interacts with each other extensively, meanwhile, each of them possesses the related two‐domain (A‐domain: α‐helical region; B‐domain: β‐rich region) structure, whose structural similarity causes pseudo‐twofold symmetry in whole GINS architecture (Figure 2C).^78^

In eukaryotic GINS, Psf1 only has an intact A‐domain, yet B‐domain is invisible in the crystal lattice, even though the similar B‐domain of Psf1 to the three other subunits via sequence alignment. Some reports indicated that the complementarity of the B‐domain into Psf1 disturbs GINS packing, which implies an essential role in CMG formation and Cdc45 binding.^79^ However, the Psf3 B‐domain is widely considered to interact with the MCM complex, strengthening the MCM3–MCM5 interface.^78^

In the CMG complex, Cdc45 cooperates with the MCM2‐7 complex to shut down the MCM2–MCM5 gate, which is crucial for ATPase site forming and CMG translocation on ssDNA. Cdc45 possesses a distinct helical motif, which is proximal to the catalytically active domain of polε. N‐terminus of polε crosslinks with Cdc45 on the tip of the protrusive helix of Cdc45, indicating Cdc45 impacts on CMG helicase and polε polymerase activity.^80^

### 3.2 PCNA and its binding proteins/enzymes

Eukaryotic sliding clamp protein, PCNA, is a ring‐shaped homo‐trimer with each subunit containing two domains, which presents a pseudo‐six‐fold symmetry pattern. Each subunit of eukaryotic PCNA is formed from two independent and semblable folded domains, which is ultimately confirmed by X‐ray crystal structure analysis. PCNA could be roughly separated into two domains, domain A and domain B, connected by an extended β sheet across the interdomain frontier. Moreover, a flexible linker concatenates two domains are named the interdomain connector loop. The assembled pattern among three subunits performs end to end structure, precisely as one domain A connects with the adjacent subunit's domain B (Figure 2D).^81^, ^82^

Due to its essential role in DNA replication, PCNA embraces the DNA and travels along it, conducting for DNA polymerases and DNA replication proteins. DNA could cooperate with three equivalent sites of PCNA since its symmetry patter. PCNA sliding along DNA counts on its basic residue interactions with the phosphates of DNA, which promoting the rotation of PCNA around the DNA. One convincing model supports that PTMs of PCNA alter its positive charges on the inner side, leading to unconscionable movement. Thermal and chemical denaturation researches demonstrated that human PCNA is much more unstable than *S. cerevisiae* homolog though they share the homogeneous three‐dimensional structure. Furthermore, human PCNA performs tough backbone dynamics, especially at helix of ring inner surface. Due to the highly dynamic and plastic property, PCNA evolves as platform to facilitate interacting with multiple proteins.^83^, ^84^

A huge collaborative network of proteins engages for high fidelity DNA repair and accurate DNA damage repair. PCNA is regarded as entire hub in DNA replication that interacts with abundant proteins involved in multiple DNA‐related processes. By occasion of homo‐trimer shape of PCNA, three identical pockets could cooperate distinct partners simultaneously and coordinate various functions spatiotemporally. Numerous PCNA‐interacting proteins (PIP) interact with PCNA via their PIP box. A typical consensus amino acid sequence of PIP motif is (Q‐x‐x‐(I/L/M)‐x‐x‐(F/Y)‐(F/Y)).^83^

The PCNA ring has three independent PIP‐box binding sites with three distinct ligands for binding proteins. To secure normal replication, three promoters of the PCNA trimer convene DNA ligase I, polδ, and FEN1 simultaneously to ensure stable Okazaki fragment synthesis. Constitutive complex has been demonstrated in yeast called the “PCNA tool belt,” which could be modulated by diverse PTMs. FEN1 interacts with PCNA via its canonical PIP box exhibiting lower affinity, while increasing the affinity by replenishing 20‐residue long PIP fragments.^81^ These observations indicate that PIP box of proteins mediates their interaction affinity to PCNA, which is also modulated by PTMs. Thus, targeting such a binding site may interfere with DNA replication and DNA damage repair, thereby serving as attractive targets for cancer therapy.

Indispensable DNA replicative polymerases are required for DNA synthesis with high efficiency and accuracy. The general architectures of DNA polymerases present right‐hand aspect with three main functional domains, which also contain exonuclease activity site for proofreading. Eukaryotic DNA replication primarily depends on three B‐family DNA polymerases: polα, polδ, and polε. Polε and polδ are chiefly for high accurate DNA synthesis on the leading and lagging stands via interaction with PCNA, respectively. All eukaryotic replicative polymerases contain two conserved motifs with cysteines (CysA and CysB), which was regard as Zn‐finger motifs originally. Except for detectable PIP box sequence in polδ, CysA motif could also directly interact with PCNA to promote efficient loading and synthesis of DNA.^31^ However, little is known about how pol ε with PCNA in mammals.

Insight into the general architecture of the replication proteins assists us in clarifying more accurate molecular regulatory mechanisms. Due to the complex interaction network, spontaneous or revulsive mutations of MCM2‐7 complex disturb the normal biological processes such as DNA replication, cell proliferation, and DDR.^57^, ^85^ Moreover, MCMs load onto DNA via particular binding domains, thus it is possible to interrupt the chromosome remodeling through interfering these specific domains.^26^, ^86^ PTMs of replication proteins in diverse residues distributed in different domains may present a special effect due to their topological alteration in positive or negative patterns. Nevertheless, the precise regulatory for how PTMs in different domain affecting downstream processes is still unclear.

## 4 THE REPLICATION STRESS RESPONSE

DNA is constantly threatened by various DNA damage stimulus including ultraviolet (UV) light, ionizing radiation (IR), biochemical reagent, which disrupting normal DNA replication and leading to RS.^87^, ^88^ The RS leads to replication fork stalling and even collapsing if the stress cannot be solved immediately.^89^ RS‐induced mitotic abnormalities can activate DNA damage repair pathway and result in activation of oncogenes.^88^, ^90^ Mutually, activation of oncogenes aggravates RS and genomic instability in human cancer cells.^91^

**FIGURE 3:**
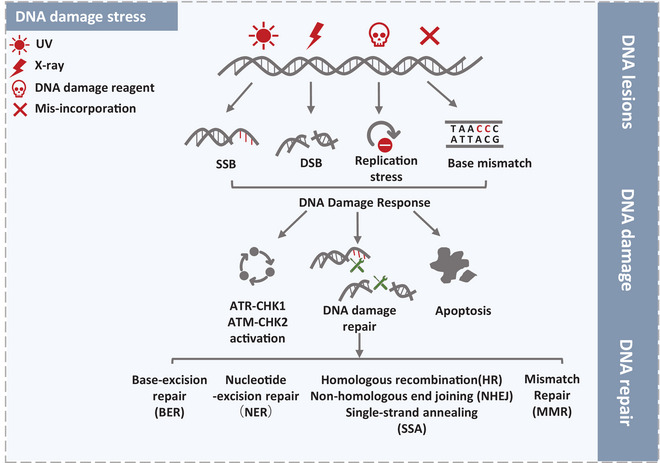
DNA damage response framework. DNA is constantly threatened by various DNA damage stimulus including ultraviolet (UV) light, ionizing radiation (IR), biochemical reagent, which disrupting normal DNA replication and leading to single‐strand break (SSB), double‐strand break (DSB), replication stress (RS), and base mismatch. DNA damage triggers sequential cascade reactions promoting cellular survival, including DNA damage repair and cell cycle checkpoint activation. Severe DNA damage may ultimately result in cell death via apoptosis. DNA repair pathway fixing damage sites is subject to the particular DNA damage types. Nucleotide excision repair (NER) is required to fix the UV light‐induced SSBs and bulky lesions. Abnormal DNA bases‐ and oxidative damage‐induced intermediates are commonly repaid by base‐excision repair (BER), whereas correct insertion loops are repaired by mismatch repair (MMR). Classic pathways to repair DSBs are homologous recombination (HR) including single‐strand annealing (SSA) and non‐homologous end‐joining (NHEJ).

If the RS cannot be fixed immediately, the replication fork will collapse thus causing DNA strand breaks.^92^ To ensure ordinary cellular events against stalled replication forks, cells harbor multiple DDR pathways to preserve genomic integrity.^93^ DNA repair pathway fixing damage sites is subject to the particular DNA damage types. In general, nucleotide excision repair (NER) is required to fix the UV light‐induced single‐strand breaks (SSBs) and bulky lesions.^94^ Abnormal DNA bases‐ and oxidative damage‐induced intermediates are commonly repaid by base‐excision repair (BER), whereas correct insertion loops are repaired by mismatch repair (MMR).^95^, ^96^ The most lethal and fearful damage type is IR‐ or chemically induced double‐strand breaks (DSBs). Classic pathways to repair DSBs are homologous recombination (HR) and nonhomologous end‐joining (NHEJ).^97^, ^98^ In addition, cell cycle checkpoint activation is also regarded as a vital DDR pathway, which includes Rad3‐related serine/threonine kinase (ATR)‐checkpoint kinase 1 (CHK1) and the ataxia telangiectasia‐mutated serine/threonine kinase (ATM)‐checkpoint kinase 2 (CHK2) pathway (Figure 3).^99^

Genomic instability is the hallmark for cancers, which is related with massive unsolved DNA damage. Based on the characteristics of cancer cells, DNA‐damaging chemotherapy is widely applied clinically even though accompanied by severe side effects to normal tissues. Given the elementary function of the DDR, DDR‐target therapy has a putative role to intercept cancer cells’ rational response through combination treatment to patients lacking specific DDR functions. Apparently, probing into the mechanisms of DNA damage repair in cancers might be an absorbing strategy for cancer therapeutic target. Since interrelated relationship between DNA replication and DDR, multiple crucial DNA replication factors are involved in DDR including MCM proteins, CMG complex, and PCNA. Intriguingly, multifunctional roles of these proteins are optimal target for cancer treatment.

RS blocks the routine DNA replication and sticks normal cell cycle, activating the cell cycle checkpoint mechanism.^100^ Since stalled replication fork forms the exposed ssDNA, RPA primarily recognizes naked ssDNA to protect it against breakage. Numerous evidences revealed that RPA serves as the most frequently responsive protein after DNA damage or during DNA repair. RPA‐coated ssDNA then unites to recruit ATR via its partner protein ATRIP (ATR‐interacting protein).^101^ Subsequently, ATR activation elicits cell cycle checkpoints and stabilizes the replication fork via phosphorylating its downstream effector kinase CHK1, further preventing damaged DNA from entering mitosis. ATR activity is also stimulated by DNA topoisomerase 2‐binding protein 1 (TopBP1), promoting its role in phosphorylating the substrates.^102^ Numerous studies indicated that the ATR‐CHK1 pathway mainly prevents S phase progression and further mediates DNA damage repair.^103^, ^104^ The function of ATR may be interrupted by numerous factors such as MCM7.^105^ Partial depletion of MCM7 directly leads to UV‐induced ATR activation defect.^106^ C17orf53 is one of the uncharacterized genes involved in ATR response.^107^ Some studies characterize that C17orf53 protein might interact with RPA1 and MCM8‐9 to regulate DNA replication and respond to DNA damage.^108^ The collapsed replication fork generates DSBs, which stimulating the DDR processes, indicating a tight relationship between DDR and DNA replication.^109^

As the MCM paradox query, abundant amounts of MCM2‐7 are exciting in most growing cells, whereas only a tiny proportion of these are used for DNA replication. Several striking outcomes have been revealed that redundant MCM proteins may serve as “backups” to ensure adequate dormant replication origins activating when suffering RS, such as in the presence of aphidicolin.^110^, ^111^ Furthermore, knockdown of MCM2‐7 increases the frequency of chromosome breaks, thus causing cells hypersensitive to RS in eukaryotes.^112^ In *Drosophila*, depleting MCM2 does not affect cell growth rate, whereas partial reduction of MCM2 decreases the number of spendable origins.^113^ In contrast, knockout of MCM7 activates checkpoint signaling in human cancer cells, prohibiting their unbitted DNA replication, which may act as the potential target for cancer treatment.^114^ Some reports also support that partial depletion of MCM2‐7 in HeLa cells does not show any noticeable impact on cell viability, whereas resulting in lethally hypersensitive to hydroxyurea (HU).^112^ Meanwhile, deletion of MCM5 also could not effect cell proliferation but makes cervical cancer cells vulnerable to RS such as HU or aphidicolin.^115^ These findings prove that excess MCM2‐7 proteins safeguard the cells against replicative stress by licensing dormant origins.

Bai et al.^116^ demonstrated that chronic RS lessened MCM2‐7 expression via a p53‐mediated manner. During exposure to low‐level RS, MCM proteins are gently decreased accordance with RNAi‐related gene silencing. The microRNA (miRNA)‐34 family targets MCM5 directly, causing descending expression of other MCM proteins and negatively regulating cell cycle progression when overexpression of these miRNAs.^116^ The eukaryotic whole‐genome analysis investigated MCM4 N‐terminal serine/threonine‐rich domain (NSD) segments combined with Rad53, Sld3, and Ddf4, to activate origin and promote replication progression to respond to RS.^117^, ^118^

The ATM and CHK2 kinases are critical regulators of double‐strand DDR. ATM activation requires the MRN (Mre11–Rad50–Nbs1) DSBs sensor complex that processes DNA ends and ATM to broken DNA molecules.^119^

The Bloom syndrome DNA helicase (BLM) is part of HR to maintain chromosome stability and promotes DNA replication after repair of DNA damage.^120^, ^121^ Shastri et al.^122^ identified BLM helicase interacts with MCM6 to resist HU‐induced RS just in S‐phase and keeps the routine DNA replication. In contrast, BLM–MCM6 is needed for cell survival under pyridostatin (RR82) induction in S‐phase, suggesting the BLM–MCM6 complex partakes in DNA replication and responds to DNA damage in eukaryotes.^122^, ^123^ Since phosphorylation of BLM shows an ATM‐dependent manner, it provides us with a possible that BLM–MCM6 complex may be regulated by ATM in DNA damage repair. Moreover, Fanconi anemia (FA) complementation group D2 (FANCD2) can directly connect to MCM2‐7 complex upon RS, thereby preventing pathological replication structure's accumulation^124^, ^125^ Naturally, FANCD2 has closely relationship with ATM, indicating ATM indirectly modulates the MCM proteins in answer to DNA RS and DNA damage.

Substantive results revealed that MCM8 and MCM9 play a vital role in HR repair as MCM8‐9 complex.^126^ Lee et al.^127^ found that incapable mutation of MCM8‐9 complex could not recognize MRN complex, leading to degressive HR efficiency. Moreover, some research proved that the depletion of MCM9 is attributed to reduced proliferation, which may be modulated by ATM‐CHK2 pathway. The MCM‐binding protein (MCMBP) is considered as a chaperone of MCM proteins to assist dynamic assembly of the MCM2‐7 hexamer and promotes MCM8‐9 for HR repair. MCM proteins connecting with MCMBP is essential for maintaining the pool of functional MCM2‐7 hexamers.^128^, ^129^

**FIGURE 4:**
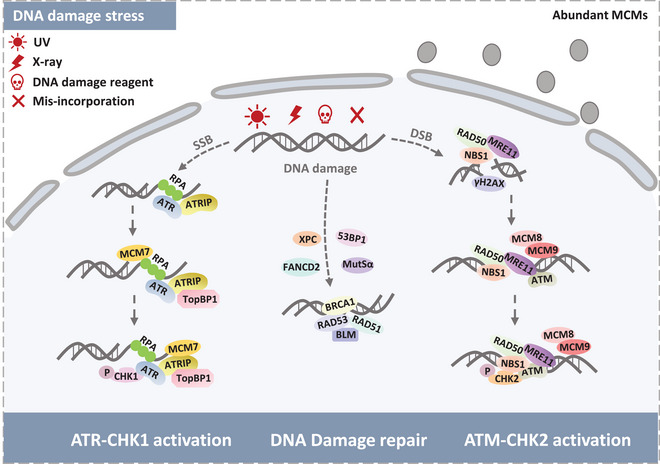
MCM proteins in response to DNA damage. RPA recognizes DNA damage‐induced single‐strand DNA (ssDNA) forming RPA‐coated ssDNA. RPA‐coated ssDNA recruits ATR via ATR‐interacting protein (ATRIP). ATR interacts with MCM7 and TopBP1 to activate CHK1 phosphorylation. Activation of ATR‐CHK1 could further lead to DNA repair activation. MCM proteins act as an intermediary in DNA repair and DNA checkpoint reactions. MRE11–RAD50–NBS1 (MRN) complex recognizes double‐standard break sites (DSBs) and recruits γ‐H2AX to DSBs. MCM proteins, especially MCM8–MCM9, are recruited by the MRN complex and cooperate with ATM to activate CHK2 phosphorylation leading to DSBs repairs such as HR and NHEJ. Multiple DNA repair proteins are recruited to the damage sites to perform distinct repair pathways, such as XPC, BRCA1, 53BP1, and so on.

MCM10, components of the replication fork, loads to DNA after MCM2‐7 complex settling down. MCM10 associates with Dna2 may function on the lagging strand during DNA replication, while Dna2 physically interacts with ATM at DNA damage sites. These complicated molecular connections imply the MCM10 potential function in stalled replication fork and DNA damage area.^130^, ^131^ One possible explanation for this circumstance is that the MRN complex stabilizes replisomes at stalled forks and recruits multiple factors to fix the predicament.^132^ Therefore, MCM10 cooperating with DSB repair proteins could exhibit one direct role of MCM10 in mediating DSB repair (Figure 4).

In conclusion, abundant MCM proteins act as a reserve to safeguard the DNA replication under DNA RS, nay, interact with multiple DNA damage factors to perform the DNA damage repair via ATR–CHK1 and ATM–CHK2 pathways. More stirring, numerous studies revealed that MCMs are phosphorylation substrates of ATM and ATR. Thus, it is remarkable to clarify how the PTMs of MCMs modulate the DNA damage repair pathway.

## 5 PTMS OF PROTEINS TARGET DNA REPLICATION AND DNA DAMAGE IN DISEASE

It is conceivable that the replication proteins’ dynamic status is regulated by known kinases such as ATM and ATR, whereas these proteins also undergo additional regulatory mechanisms. Substantive publications have revealed distinct replication proteins exercise unpredictable functions achieved by diverse PTMs.^11^ Such PTMs contain phosphorylation, ubiquitination (Ub), small ubiquitin‐like modifier (SUMOylation), O‐N‐acetyl‐d‐glucosamine (GlcNAcylation), and acetylation. Recent results revealed that PTMs of these proteins contribute to DNA replication and DNA damage repair, which also could be potential therapeutic target for tumors.^133^

### 5.1 MCMs phosphorylation

Individual MCMs are subject to phosphorylation in a cell cycle‐specific manner, which may be consistent with their cell cycle‐specific functions. Due to various kinase types, phosphorylation of MCM proteins undergoes distinct regulatory mechanisms. Moreover, exceptional phosphorylation of MCMs might disrupt DNA replication progression, further causing DNA damage and leading to diseases or cancers (Table 1).^134^

**TABLE 1:** Summary of the MCMs modification in response to DNA replication and DNA damage

Modification type	Mediator	Substrate	Function	Reference
Phosphorylation	CDK	MCM2‐S139	Promote chromatin loading	^135^
		MCM3‐S112	Promote MCM2‐7 incorporation	^138^
		MCM3‐S711	Cell cycle regulation	^135^
		MCM3‐T722	Promote chromatin loading	^136^
Phosphorylation	CDK	MCM4‐T7, T9, S32, T110	Decrease the binding of MCM to DNA	^140^
		MCM4‐S3, S32	Activation cell cycle checkpoint	^141^, ^142^
		MCM7‐S121	Promote chromatin loading	^137^, ^139^
		MCM7‐S365	Cell cycle regulation	^137^, ^139^
	DDK	MCM2	Interact with CDC45 and GINS	^143^
		MCM2‐S27, S41, S139	Maintain genome integrity	^144^
		MCM2‐S164, S170	Proper response to DNA damage	^145^, ^146^
		MCM4‐NSD	Cell growth and S phase progression	^147^, ^148^
		MCM6‐NSD	Cell proliferation	^147^, ^148^, ^149^
		MCM10	Facilitate double hexamer separation	^23^, ^150^
	ATR	MCM2‐S108	Response to DNA damage	^135^
		MCM6‐S13	Response to DNA damage	^153^
	ATM	MCM3‐S535	Cell cycle checkpoints activation	^151^
		MCM3‐S725, S732	Response to DNA damage	^152^
	ATR/ATM	MCM10	Response to DNA damage	^154^
Ubiquitination	KEAP1	MCM3	Preserve genome stability	^156^
	HERC2	MCM6	Preserve genome stability and DNA repair	^157^, ^158^, ^159^
	UBE3A	MCM6	Preserve genome stability and DNA repair	^157^, ^158^, ^159^
	CDC49/p97	MCM7	ICL repair	^160^, ^161^
	BRCA1	MCM7	ICL repair and HR	^162^, ^163^
	SCF^Dia2^	MCM2‐7	CMG helicase disassembly	^164^
	CUL^LRR1^	MCM7	Preserve genome stability	^165^, ^166^
	TRAIP	MCM7	CMG helicase disassembly, ICL repair	^167^, ^168^, ^170^
	CUL4^DDB1^	MCM10	Preserve genome stability	^171^, ^172^
SUMOylation	Slx5/Slx8	MCM2‐7	In response to replication stress	^176^, ^177^
	Mms21	MCM2, MCM3	Preserve genome stability	^180^, ^181^, ^182^
	Ulp1/Ulp2	MCM4, MCM7	Preserve genome stability	^179^
	Siz1, Siz2	MCM2, MCM3, MCM4, MCM5, MCM7	Preserve genome stability	^178^
		MCM2, MCM3, MCM4, MCM7	In response to cytotoxic stress	^173^, ^174^, ^175^
		MCM10	Preserve genome stability	^183^
Acetylation	HBO1	MCM2	Preserve genome stability	^186^, ^187^, ^188^
	MCMAP	MCM3	DNA replication	^185^
	p300	MCM10	Stabilize genome integrity	^189^
	SIRT1	MCM10	Stabilize genome integrity	^189^
O‐GlcNacylation	OGT	MCM2‐7	Preserve genome stability	^191^
Methylation	KMTS	MCM2‐7	Response to heat stress	^192^

#### 5.1.1 CDK/DDK‐mediated phosphorylation

Cyclin‐dependent kinases (CDKs) and their regulatory proteins cyclin are main protein kinases to modulate the progression from G1 into S phase and from G2 into mitosis. Thus, different CDKs–cyclin assemblages phosphorylate MCMs to influence the cell cycle progression or DNA damage repair pathway.

MCM2 and MCM3 are generally phosphorylated by CDK2 at Ser‐139 and Ser‐711 in eukaryote, respectively.^135^ MCM3 phosphorylation at Thr‐722 promotes MCM complex chromatin loading, which is medicated by cyclin E/CDK2.^136^ What is more, cyclin E/CDK2‐mediated MCM7 phosphorylation at Ser‐121 in HL‐7702 cells also facilitates its chromatin loading and normal mitosis.^137^

MCM3 Ser‐112 is phosphorylated by CDK1, promoting the connection among MCM subunits and MCM3 chromatin loading in U20S cells.^138^ Alternatively, ineffective MCM3 phosphorylation may impair MCM2‐7 helicase activity, resulting in S phase delay and activating S phase checkpoint, ultimately causing a turbulent cell cycle. When stalled replication fork activated cell cycle checkpoint, abundant MCM proteins, especially MCM3 and MCM7, are assembled at damage sites to block the S phase entry. Uniformly, some research demonstrated that overexpression of the wild‐type MCM7 resulted in S phase block. MCM7 Ser‐121 is strongly phosphorylated by cyclin B/CDK1, whereas Ser‐365 is phosphorylated by CDK2.^137^, ^139^ These findings indicate that phosphorylation of MCM7 interferes its DNA loading ability. In contrast, dephosphorylation of MCM7 protects the cell cycle when confronting RS. Additionally, CDK1 phosphorylates MCM4 at Thr‐7, Thr‐19, Ser‐32, Ser‐88, and The‐110, while Ser‐3 and Ser‐32 are phosphorylated by CDK2. These modifications decrease the ability of MCM2‐7 to load onto DNA, avoiding re‐replication during mitosis.^140^ More obviously, MCM4 is phosphorylated by CDK under HU and UV irradiation, which is critical to stimulate cell cycle checkpoint activation.^141^, ^142^

In addition, another DNA replication‐associated kinase, Dbf4‐dependent kinase (DDK), also is essential for the phosphorylation of the MCM2‐7 complex. DDK‐mediated phosphorylation of MCMs induces a conformational change, therefore impacting the connection with other DNA replication factors. The observation indicated that DDK‐dependent MCM2 phosphorylation dissociates from MCM5, unfolding the MCM2‐7 hexameric to prevent DNA re‐replication. Electron microscopy analysis revealed that the interaction of MCM2‐7 with CDC45 and GINS promote MCM2–MCM5 gap blocking.^143^

Tsuji *et al.* identified three DDK‐dependent MCM2 phosphorylation sites (Ser‐27/41/139), both in vivo and in vitro. Deactivation mutation of MCM2 (Ser27/41/139‐Ala27/41/139) blocks DNA replication and causes RS, which suggests that DDK‐mediated phosphorylation of MCM2 closely regulates DNA replication.^144^ In addition, other studies revealed that phosphorylation of MCM2 by DDK is critical for MCM2‐7 ATPase activity in vitro. Previous studies showed that phosphorylation of *S. cerevisiae* MCM2 by DDK at Ser‐164 and Ser‐170 is crucial for a proper response to DNA damage.^145^ Further research demonstrated that the phospho‐deficient mutation of MCM2 (Ser164‐Ala, Ser170‐Ala) increased sensitivity to HU and base analog 5‐fluorouracil (5‐FU) as spontaneous mutation rate, which expressly revealed DDK‐mediated MCM2 phosphorylation modulated MCM2‐7 activity and preserved genome stability in response to replicative stress.^146^ On the other hand, other research pointed to the NSD of MCM4 is the target DDK to promote S phase progression.^147^ Taken together, DDK‐mediated phosphorylation of MCM2 and MCM4 serves as a critical point in modulating the MCM2‐7 complex dynamic motion and protecting the genome integrity.

Except for MCM2 and MCM4, MCM6 has an unstructured N‐terminal domain containing certain DDK target sites, and is phosphorylated by DDK in vitro.^148^ Importantly, MCM4 and MCM6 NSD are phosphorylation in G1, S, and G2/M phase, which are vital for cell viability. Notably, inhibition of the MCM4/6 phosphorylation leads to additional growth defects, further causing genome instability.^149^ Previous research demonstrated that DDK associated with MCM10 in vitro, which is consistent with an earlier finding in *Schizosaccharomyces pombe*.^150^ MCM10 also interworks with MCM2‐7 to facilitate double hexamer separation, which is influenced by CDK and DDK‐mediated phosphorylation.^23^

#### 5.1.2 ATM/ATR‐mediated phosphorylation

According to the above description, MCM proteins are involved in the ATM/ATR signaling pathways to perform their DNA damage repair functions. In addition, ATM and ATR also serve as the master kinase to phosphorylate MCMs, stabilizing the DNA replication fork and actives cell cycle checkpoints. Cortez et al.^151^ found that ATM phosphorylates MCM3 Ser‐535 under IR, whereas multiple DNA damage agents could cause ATR‐dependent MCM2 phosphorylation, such as radiation exposure and chemical reagents. Some reports also revealed that ATR‐mediated MCM2 is phosphorylated without stimulating DNA damage.^135^ Further, ATM contributes to MCM3 C‐terminal Ser‐725 and Ser‐732 phosphorylation upon unstable condition. However, this phosphorylation may not cause MCM2‐7 complex conformational change.^152^ Wagner et al.^153^ found that MCM6 Ser‐13 was a novel putative ATR target site in answer to RS. UV irradiation disturbs DNA replication progression since MCM10 proteolysis in human cells. UV‐induced MCM10 degradation might be rescued by interfering with ATR/ATM inhibitor and CHK1 inhibitor, indicating that ATR and CHK1 kinase modulate its downregulation.^154^ Taken together, ATR/ATM‐mediated MCMs phosphorylation is crucial for responding to DNA RS and DNA damage repair.

In summary, MCMs can be phosphorylated by multiple kinases, which is critical to maintain the genome integrity from DNA RS and respond DNA damage. However, the mechanistic details for distinct MCM subunits phosphorylation triggering downstream repair components still need to be clarified. Further studies are essential to elucidate undiscovered and putative phosphorylation sites, which is essential for insight into selective approaches to repair DNA damage.

### 5.2 MCMs Ubiquitination

Protein Ub is a well‐known pathway for target protein degradation. Otherwise, protein Ub also modulates multiple cellular biological processes such as DNA replication, cell cycle checkpoint activation, and DNA repair. Mass spectrometry (MS) results showed that all the MCM proteins in eukaryotes are ubiquitinated in human cells. Of those, MCMs are ubiquitinated by diverse E3 ligases when cells are threatened by DNA damage or RS (Table 1).^155^

The Kelch‐like ECH‐associated protein 1 (KEAP1) is one crucial candidate of the Cullin3 (CUL3)–RBX1 E3 ligase complex, which ubiquitinates MCM3 in actively proliferating cells.^156^ KEAP1‐mediated MCM3 Ub regulates cell cycle progression and genome stability by controlling the MCM2‐7 complex helicase activation. Actually, KEAP1 itself serves as the crucial component in response to oxidative stress, which may be achieved through MCM2‐7 complex chromatin loading.

Recent Ub proteomic analysis revealed that ubiquitin protein ligase E3A (UBE3A) could interact with HERC2 and MCM6 with unknown functions.^157^ Apparently, HERC2 is a crucial DNA damage repair factor participating in HR repair at DSB sites. In addition, HERC2, with RNF8, has been shown to promote translesion synthesis (TLS) at stalled replication forks.^158^, ^159^ Thus, not far to seek, UBE3A‐mediated MCM6 Ub may interact with HERC2 to keep the chromosome stable and further play a role in DNA repair.

During DNA replication termination, CDC49/p97 complex targets polyubiquitinated MCM7 to disengage CMG complex, thus terminating DNA replication.^160^, ^161^ George et al.^161^ supported that polyubiquitylation of MCM7 has a modest effect to interstrand cross‐links (ICLs) repair, which suggests that MCM7 proteasomal degradation may play a more active role in response to DNA damage. Moreover, illustrious HR repair‐associated factor BRCA1 serves as upstream of MCM7 Ub.^162^ BRCA1 recruits additional E3 ligases to promote MCM proteins and CMG complex Ub.^163^ It is necessary that helicases remove from the damaged DNA after accomplishing recovery. During ICL repair, BRCA1‐mediated CMG Ub assists their disassembly, positioning a distinct regulatory signal to ensure unloading initiation. Thus, Ub‐mediated MCMs unloading provides an appropriate occasion to resolve RS.

The best‐characterized E3 ligase comes from *S. cerevisiae*, cullin 1 ligase SCF^Dia2^, drives CMG ubiquitylation to perform CMG helicase disassembly. However, CMG depolymerizing has various pathways during eukaryotic evolution.^164^ Subsequent works indicated that cullin 2 ligase CUL2^LRR1^‐mediated MCM7 Ub is essential to preserve genome stability during DNA replication termination both in yeast and in human cells.^165^, ^166^ Further work indicated that two crucial E2 enzymes, UBE2R1/R2 and UBE2G1/G2, connect with CUL2^LRR1^ to extend a polyubiquitin chain on MCM7.^165^ Ub of MCMs departs from chromatin due to their topological alternation, which may form a functional MCM2‐7 hexamer in their de‐Ub pattern.

Deng et al.^167^ suggested that tumor necrosis factor receptor‐associated factor‐interacting protein (TRAIP) acts as ubiquitin ligase associating with the CMG replisome, thus triggering replication fork collapse. Previous work indicated that TRAIP is crucial in regulating normal cell cycle to keep genome stability in eukaryotes.^168^ However, Favrizio et al.^166^ found that TRAIP‐mediated Ub of MCM7 triggers CMG disassembling in mouse embryonic stem cells. ICL‐induced DNA replication stalling is repaired by endonuclease 8‐like protein 3 (NEIL3) glycosylase and FA pathway.^169^ Wu et al.^170^ identified stalled replication fork triggers TRAIP‐mediated MCM7 Ub to participate in ICL repair. TRAIP‐mediated MCM7 Ub causes distinct ICL‐repair pathways, NEIL3 recognized short ubiquitin chains to cleave directly, while long ubiquitin chains recognized by p97 complex to trigger FA pathway.

In *S. cerevisiae*, MCM10 is mono‐ubiquitinated at two distinct lysine sites, subsequently, interacts with PCNA.^171^ Expression level of MCM10 is precisely mediated by the CUL4^DDB1^ complex.^172^ De‐Ub of MCM10 results in hypersensitive to HU owing to dysregulation of the interaction between MCM10 and PCNA.

In summary, K48‐linked MCM7 degradation leads to disassembly of the MCM2‐7 complex, which is critical in DNA replication termination. However, BRCA1‐mediated Ub of MCM7 takes part in HR and ICL repair but not in replication termination. It provides us a novel sight that Ub in different MCM subunits or various sites performs distinct functions. It also possible that specific E3 triggers distinct Ub‐chains to ubiquitinate MCMs for degradation or activation pattern.

### 5.3 MCMs SUMOylation

SUMO is a protein modifier that plays crucial roles in a wide range of cellular processes, making it essential for the viability of most eukaryotes. SUMOylation is a multistep process modulated by specific E1, E2, and E3 enzymes, like Ub. Compared with Ub, SUMOylation of proteins do not mediate their degradation, modulating their subcellular compartmentalization and reinforcing their stability (Table 1).^173^, ^174^

Previous studies showed that DNA alkylating agents stimulated SUMOylation of MCMs except for MCM3 and MCM7. However, MCM2, 3, 4, and 7 were SUMOylated in response to heat shock in human cell, indicating that MCM SUMOylation may modulate cells against cytotoxic stress.^175^

The SUMO‐target ubiquitin ligase Slx5/Slx8 in *S. cerevisiae* are crucial in modulating DNA repair via SUMOylation repair factors.^176^ Coincidentally, Slx5‐based proteomic research revealed that MCM2‐7 complex may be as potential substrates of Slx5/Slx8. These data suggest the Slx5/Slx8‐mediated SUMOylation of MCM2‐7 may take effect during DNA replication and DDR.^177^

SUMO modification of MCM3 at K767 and K768 may work together to directly or indirectly promote MCMs loading onto chromatin. Site‐specific mutagenesis of MCM3 K767/768 leads to MCM2‐7 complex disassembly and CMG complex collapse, delaying the chromosomal DNA replication and leading to genome instability. Uniformly, factitious de‐SUMOylation of MCM3 may generate spontaneous DSBs due to incomplete DNA replication, which is quite lethal to cells. ^175^

*S. cerevisiae* harbors three SUMO E3 ligases: Siz1, Siz2, and Mms21, which are necessary for controlling intracellular activities.^178^ Except for SUMO E3 ligases, SUMOylation is also modulated by SUMO isopeptidase Ulp1 and Ulp2, which performing de‐SUMOylation effect. Ulp2 is inutile for cell viability but necessary for the accumulation of poly‐SUMO chains.^179^ de Albuquerque et al.^180^ found that loss of Ulp2 aggravates SUMOylation of MCM4 and MCM7, while partially downregulating MCM6 SUMOylation. Mms21, but not Siz1 and Siz2, mediates SUMOylation of MCM3 under HU stimulation, suggesting that Mms21‐dependent SUMOylation of MCM3 might contribute to regulating DNA replication and respond to DNA RS.^180^ Siz1/Siz2‐mediated SUMOylation of MCMs has been detected in unperturbed cells, whereas Mms21 preferentially interacting with MCM2 and MCM3.^181^ Wei and Zhao found that SUMOylation of MCMs exhibits preference for chromatin‐bound MCM subunits including MCM4, MCM6 and MCM7. SUMOylation of MCM proteins leads to decreased CMG protein levels and inhibits DNA replication initiation.^182^

Tian et al.^183^ identified a germline variant rs2274110 in MCM10 that confers an inferior survival of esophageal squamous cell carcinoma (ESCC) patients. This functional variant can increase MCM10 SUMOylation resulting in aberrant overexpression, substantially facilitating ESCC progression via fueling DNA over‐replication and genomic instability. These findings underline that PTMs of MCM proteins may serve as potential therapeutic targets in tumor treatment.^183^

### 5.4 MCMs acetylation

Lysine acetylation is a widespread and versatile protein PTM. Indeed, nonhistone protein acetylation is deemed as a key regulatory component in multiple biological processes such as DNA replication, DNA damage repair, autophagy, and metabolism. Several studies revealed that MCM proteins are substrates for acetylation (Table 1).^184^

MCM3AP acts as an acetyltransferase to acetylate MCM3, which promotes the translocation of MCM3 from the cytoplasm into the nuclei. Moreover, MCM3AP‐mediated acetylation of MCM3 can inhibit DNA replication.^185^

HBO1 complexes belong to the MYST family and are major acetyltransferases aiming for histone H4 acetylation in vivo. More recently, HBO1 was deemed to modulate the replication origin of Kaposi's sarcoma‐associated herpes virus. These functional interactions implied a putative function of HBO1 in pre‐RC formation and replication licensing.^186^ Lizuka et al.^187^ demonstrated that HBO1 significantly acetylates DNA replication‐associated proteins, such as ORC2, MCM2, CDC6, and Geminin. HBO1‐mediated MCM2 might regulate the initiation of DNA replication.^187^ During DNA replication initiation step, recruitment of HBO1 to origin by Cdt1 is required for MCM2‐7 complex loading in human cells, which may stabilize the interaction of MCM complex with chromatin.^188^ It provides a novel insight that HBO1 may acetylate MCMs to perform conformation alternation, further modulating the DNA replication or DNA damage repair.

SIRT1 is a histone deacetylase that has been implicated in containing chromatin structure and DNA repair, serving as a crucial guard to maintain genomic stability. Samuel et al.^189^ demonstrated that deacetylation of MCM10 by SIRT1 is one of the vital regulatory events in preserving genome stability. Moreover, MS and biochemical analysis indicated that twelve lysine residues of MCM10 acetylated by p300 are involved in DNA binding.^189^ These results indicated that the dynamic balance of MCM10 acetylation has to be tightly regulated for proper fork initiation and stable genome integrity.

### 5.5 Other PTMs of the MCM proteins

Protein O‐GlcNacylation is involved in multiple biological processes, especially in stress response. O‐GlcNacylation of proteins is catalyzed by O‐GlcNAc transferase (OGT) to transfer the GlcNAc group onto serine or threonine residues of proteins. Reciprocally, O‐GlcNAcase (OGA) reverses these PTMs by removing the GlcNAc residue.^190^ In mammalian cells, O‐GlcNAcylation levels are dynamic alternation during the cell cycle, thus abnormal O‐GlyNAc dynamic cycling disrupts the cell cycle and causes RS. Using a mass‐tagging strategy, Leturcq et al.^191^ identified that MCM2‐7 all subunits are O‐GlcNAcylated by OGT in human cells, especially in MCM3, MCM6, and MCM7. Each subunit of MCM2‐7 complex gradually disperses in knockout OGT cells, subsequently departing from chromatin. Thus, it is tempting to speculate that O‐GlcNAcylation of MCMs assists MCM assembly and regulates dynamic balance during DNA damage and DNA replication.^191^

Lysine methylation usually occurs in histones, whereas in nonhistones in recent decades. Methylation can alter the conformation of proteins thus changing their function. Xia et al.^192^ identified that recombinant *Sulfolobus* MCM (*sis*MCM), an archaeal homolog of MCM2‐7 eukaryotic replicative helicase, is mono‐methylation by aKMT4 in vitro, which is characterized as the first archaeal lysine methyltransferase. Interestingly, MCM methylation (me‐MCM) upregulates MCM complex DNA unwinding ability, modulating their helicase activity. More intriguingly, me‐MCM also enhances heat resistance, which supports that methylation of MCM proteins also impacts protein thermal properties (Table 1).

### 5.6 PCNA Ubiquitination and SUMOylation

During DNA replication, PCNA serves as the pivot to recruit replicative polymerases polε and polδ to perform high‐fidelity DNA synthesis.^193^ When replicative DNA polymerase encounters damaged DNA, the progression of the polymerases is blocked, and the replication fork is stalled. If this problem were not be resolved, the replication fork would be collapsed, resulting in cell death. As we summarized above, the blooey replication fork activates a unique DNA repair pathway called postreplication repair (PRR), such as error‐prone translesion DNA synthesis (TLS) and error‐free template switching (TS).^194^ Specialized DNA TLS polymerases have been identified in yeast and mammalian, including polymerase η (polη), polymerase ι (polι), and polymerase κ (polκ). For instance, polη mediates efficient and precise TLS past UV‐induced thymine‐thymine CPD (T‐T CPD), whereas resulting in the high frequency of mutations in routine replication progression.^195^ Low‐fidelity TLS polymerase causes incorrect nucleotide insertion in normal replication, subsequently stimulating NER, BER, or HR pathways to fix the errors.^196^

PCNA is mono‐ubiquitinated at K164 by RAD6–RAD18 E2–E3 complex in response to replication fork stalling.^197^ Mono‐ubiquitinated PCNA increases affinity with polη, thus further promoting efficient TLS.^198^ Hence, dysregulation of replication and TLS progression cause severe genomic instability and tumorigenesis. Except for the Ub of PCNA, multiple modifications are involved in regulating PCNA functions, thereby impacting DNA replication, DNA repair, and even carcinogenesis.

Ub of PCNA undergoes regulation and control from the chromatin microenvironment by various factors. The chromatin structure could be regulated by PTMs of histone and nucleosome remodeling, which is vital for DNA replication, cell cycle, and DNA damage repair. Mutation of histone H3 and H4 disrupts DNA packaging, resulting in reduced expression of PCNA Ub under methyl methanesulfonate (MMS) or UV irradiation.^199^

RAD6–RAD18‐mediated PCNA–K164 mono‐Ub is widely known in a variety of organisms. Meanwhile, polyubiquitination of PCNA has also been observed at K164 residue in yeast and mammal cells, which is essential for inducing error‐free pathways upon damage response.^200^, ^201^ K63‐linked polyubiquitination of PCNA requires a ternary complex composed of RING‐finger E3 ligase RAD5 and Mms2–Ubc13 complex, protecting cellular DNA against genomic mutations via a TS pathway.^200^, ^202^, ^203^

RNF8 is a crucial E3 ligase in regulating histone Ub during DNA damage repair, while it is also identified as a novel E3 ligase for PCNA. Nevertheless, RNF8 cooperates with distinct E2 such as Ubc15 and Mms2 to activate mono‐Ub and polyubiquitination, respectively.^204^ Depletion of RNF8 in medulloblastoma cells significantly suppresses PCNA mono‐Ub under UV damage, which is associated with UV‐induced p53 targeting and checkpoint activation.

In response to stalling DNA replication fork, PCNA could also be modified by SUMOylation at K164 residue, which is commonly observed in yeast and mammals. Furthermore, some reports also supported that PCNA might be SUMOylated at K127 residue and K164 in response to DNA damage.^205^ SUMOylation is closely related to Ub that modulates protein coordinated interaction, whereas possibly antagonizes Ub via competing the same residue in substates.^206^ In contrast, forceful evidence supported that SUMOylation of PCNA prohibits its Ub and DNA damage repair progression, implying SUMO of PCNA functions on regulating normal DNA replication.

In *S. cerevisiae*, SUMOylation of PCNA recruit helicase Srs2 via its C‐terminus SUMO‐interaction motif (SIM), which disrupt RAD51 single‐stranded presynaptic filaments, ultimately interrupt HR progression.^207^ Furthermore, RFC is required for PCNA SUMOylation, which facilitating PCNA loading onto chromatin. SUMOylation of PCNA also cooperates with PIP, suppressing inappropriate HR.^208^ Moreover, Gali et al.^209^ revealed that SUMOylation of PCNA could decrease DSBs formation using neutral comets assay, even lower spontaneous recombination frequencies, and enhance damage resistance. Thus, impaired PCNA SUMOylation facilitates DSB formation at stalled replication fork, which highlight its role in preserving genome integrity.

One intriguing perspective supports that SUMOylation could conduct for a signal for Ub. SUMO‐targeting ubiquitin ligases (STUbLs) intervenes protein SUMOylation and Ub of SUMO segments via its SIMs. Mutation of PCNA SUMOylation site K164/K127 and RAD18 SIMs residues directly decrease PCNA Ub level, implying SUMOylation of PCNA facilitates RAD18 to target PCNA.^210^ Therefore, it points out that the SUMO and Ub crosstalk may be essential for DNA replication and DNA damage repair (Table 2).

**TABLE 2:** Summary of the PCNA modification in response to DNA replication and DNA damage

Modification type	Mediator	Function	Reference
Ubiquitination	RAD18	TLS repair	^197^, ^198^
	RAD5	Preserve genome stability and DNA repair	^202^, ^203^
	HLTF	TLS repair	^200^
	RNF8	Preserve genome stability and DNA repair	^204^
SUMOylation		Preserve genome stability and DNA repair	^205^, ^206^
		HR repair	^207^, ^208^, ^209^
	STUBLs	DNA replication and DNA repair	^210^

## 6 DNA REPLICATION, DISEASE, AND THERAPY

Dysregulation of DNA replication is one remarkable feature of cancer cells associated with tumor progression.^6^ Since DNA replication is strictly regulated by CMG complex and multiple polymerases, dysregulation of such replication factors may contribute to abnormal cell cycles causing severe consequences.^211^ Accumulating evidence suggests that aberrant expression of CMG serves as reliable diagnostic markers among some cancers.^212^ Consistently, MCMs are involved in multiple DDR pathways, such as activation of cell cycle checkpoint, which are also considered as crucial cancer therapeutic strategies.

### 6.1 MCMs in tumorigenesis and development

Genome instability is a hallmark of cancer. Conventional expression of the MCM2‐7 complex ensures routine DNA replication progression, which is a prerequisite for genome stability.^213^ Nevertheless, numerous studies have indicated that both deficiency and overexpression of MCMs are associated with cancer development. Mcm3‐deficient mouse model was used to determine the impact on gene function in hematopoietic stem cells. The results indicated that downregulation of MCM3 results in RS, further leading to fetal anemia during embryonic development.^214^ In contrast, MCM6 was overexpressed in clear‐cell renal cell carcinoma.^215^ Upregulation of MCM6 also exists in non‐small cell lung cancer and breast cancer with worse survival and higher histological grade.^216^, ^217^, ^218^ The reasons for abnormal MCMs expression remain unclear. There are two possible speculations: (a) CDK‐mediated MCM complex dissociating prevents DNA re‐replication. However, dysregulation of the cell cycle‐dependent kinase CDK permits MCMs constantly binding to each other, resulting in continuous cell division and high expression.^219^, ^220^ (b) Aberrant DNA replication license system induces abnormal DNA replication, increasing genomic instability, and carcinogenesis.^221^, ^222^ In addition, spontaneous mutation of MCMs increases chromosome elimination and DNA damage,^223^ whereas a more than twofold reduction of MCM protein expression could lead to genomic instability in *S. cerevisiae*. These findings demonstrated that MCMs are indeed involved in tumorigenesis, but the detailed mechanism is still unclear.^212^

In lung cancer, omics data of MCM2 overexpression is analyzed using the Gene Expression Omnibus database, which is associated with large tumor size, different malign degrees, and clinical stages.^224^ Using RT‐PCR analysis, except for MCM3 and MCM5, MCMs are upregulated in cervical cancer in vitro and in vivo, which are critical in tumor progression.^225^ *Spontaneous dominant leukemia* (*Sdl*) mice model is a murine model for heritable T cell lymphoblastic leukemia/lymphoma, which harbors a spontaneous mutation in Mcm4 (Mcm4^D573H^). Mcm4^D573H^ could not alter the total expression of MCM2‐7 complex, whereas it significantly promotes tumor formation.^226^ In laryngeal squamous cell carcinoma cells, knockout of MCM4 using siRNA also suppresses cell proliferation and inhibits of tumor progression.^227^

MCM7 is regarded as an extensive mark for cancer development.^228^ Some studies indicated that MCM7 genome sequence embodies a cluster of miRNAs (miR‐106b, miR‐93, miR‐25), which can downregulate the expression of oncogenes, including p21, E2F1, BIM, and PTEN.^229^ PRMT5 acts as one methyltransferase to methylate multiple proteins in histones,^230^ which is also deemed a potential target in colorectal cancer development and progression.^231^ Recent studies revealed that PRMT5 physically interacts with MCM7 in HCT8 cells, while MCM7 depletion impairs cancer cell migration and invasion.^232^ Using TCGA analysis, the expression of MCM7 enhanced by approximately 12% in ESCC. Silencing of MCM7 via siRNA significantly impaired KYSE510 cell proliferation and migration in vitro.^233^ Furthermore, miR‐214 targets overexpression of MCM5 and MCM7 in hepatocellular carcinoma (HCC) cells to inhibit cell replication and colony formation.^234^

Massive rearrangements are one of the characteristics of aggressive cancer genomes. MCM8, unclassical MCM proteins, is deemed to interrelate with chromosome rearrangement.^235^ Knockdown of MCM8 in mice diminished xenografted tumor volume, which implied the critical role of MCM8 in tumor metastasis in vivo.^236^

Despite novel and striking findings, additional investigations are still needed to be addressed. Since closely associated with tumorigenesis, MCMs can be used as precise cancer therapy via molecular targets.

### 6.2 MCMs as diagnostic and prognostic biomarkers

As mentioned above, aberrant expression of MCMs is closely related to tumorigenesis and development. Therefore, MCMs are also used as tumor biomarkers and indicators of prognosis.^237^ For example, MCM2 is regarded as a novel proliferation biomarker for oligodendroglioma,^238^ ESCC,^239^ and breast cancer.^240^, ^241^ However, the detailed interventional mechanism is different due to MCM2's diversiform role in the distinct process. Knockdown of MCM2 abolishes DNA damage in ESCC cells, interfering with DNA replication in breast cancer cells. Meanwhile, MCM2 is also suggested to be a prognostic marker for some tumors such as renal cell carcinoma,^242^ laryngeal carcinoma,^243^, ^244^ and gastric cancer.^245^, ^246^ In oral squamous cell carcinoma^247^, ^248^ and large B‐cell lymphoma,^249^, ^250^ MCM2 also nominates as a prognostic marker significantly related to malignant progression and the 2‐year survival rate of patients, respectively. In addition, abnormal expression of MCM3 reflects advanced tumor stage and metastatic status in cervical cancer,^251^, ^252^ breast cancer,^253^, ^254^ oral squamous cell carcinoma,^255^ malignant salivary gland tumors,^256^, ^257^ and HCC.^258^, ^259^ Simultaneously, based on the TCGA and GEO analysis, some reports persist that MCM4 mainly serves as a prognostic indicator for HCC,^260^, ^261^ which is relevant to poor prognosis with MCM4 overexpression pattern. Furthermore, overexpression of MCM4 may also be a diagnostic signal in esophageal cancer,^262^, ^263^ colorectal cancer,^264^, ^265^ cervical cancer,^225^, ^266^, ^267^ ovarian cancer,^268^, ^269^, ^270^, breast cancer,^56^ and gastric cancer.^271^, ^272^ In addition, upregulated expression of MCM5 is mainly related to poor prognosis and malignant status.^115^, ^225^, ^273^, ^274^, ^275^ Consistent with this notion, overexpression of MCM5 also associates with tumor stages, which appears to be potential diagnostic and prognostic markers in thyroid cancer,^276^, ^277^ ovarian cancer,^269^, ^278^, ^279^, ^280^ bladder cancer,^281^, ^282^, ^283^ and renal cell carcinoma.^284^

On the contrary, WGCNA combining with TCGA and GEO analysis identified that exceptional expression of MCM6 reflects pathologic stage of multiple tumors, serving as putative biomarkers for breast cancer,^285^, ^286^, ^287^ gastric cancer,^288^, ^289^ renal cell carcinoma,^215^, ^290^, ^291^ HCC,^292^, ^293^, ^294^, ^295^ and non‐small cell lung carcinoma.^217^, ^296^, ^297^ Meanwhile, MCM7 is widely regarded as an extensive biomarker in multiple tumor types since its overexpression pattern.^233^, ^234^, ^298^, ^299^, ^300^, ^301^, ^302^, ^303^, ^304^, ^305^, ^306^, ^307^, ^308^, ^309^, ^310^ Except for conventional MCM proteins, MCM8, MCM9, and MCM10 are also novel prognostic markers in multiple tumors. In tissues, high expression of MCM8 may act as a valuable prognostic indicator for different cancer therapy, consistent with gastric and cervical cancer.^311^, ^312^, ^313^ MCM9 and MCM10 are associated with additional tumor types such as colorectal cancer, breast cancer, ovarian cancer, and HCC (Table 3).^314^, ^315^, ^316^, ^317^, ^318^, ^319^, ^320^, ^321^, ^322^

**TABLE 3:** MCM proteins as diagnostic and prognostic biomarker

Proteins	Cancer type	Reference
MCM2	Oligodendroglioma	^238^
	Esophageal squamous cell carcinomas	^239^
	Breast cancer	^240^, ^241^
	Renal cell carcinoma	^242^
	Laryngeal carcinoma	^243^, ^244^
	Gastric cancer	^245^, ^246^
	Oral cancer	^247^, ^248^
	Large B‐cell lymphoma	^249^, ^250^
MCM3	Cervical cancer	^251^, ^252^
	Breast cancer	^253^, ^254^
	Oral squamous cell carcinoma	^255^
	Malignant salivary gland tumors	^256^, ^257^
	Hepatocellular carcinoma	^258^, ^259^
MCM4	Hepatocellular carcinoma	^260^, ^261^
	Breast cancer	^55^
	Esophageal cancer	^262^, ^263^
	Colorectal cancer	^264^, ^265^
	Cervical cancer	^225^, ^266^, ^267^
	Ovarian cancer	^268^, ^269^, ^270^
	Gastric cancer	^271^, ^272^
MCM5	Cervical cancer	^115^, ^225^, ^273^, ^274^, ^275^
	Thyroid cancer	^276^, ^277^
	Ovarian cancer	^269^, ^278^, ^279^, ^280^
	Bladder cancer	^281^, ^282^, ^283^
	Renal cell carcinoma	^284^
MCM6	Breast cancer	^285^, ^286^, ^287^
MCM6	Gastric cancer	^288^, ^289^
	Renal cell carcinoma	^215^, ^290^, ^291^
	Hepatocellular carcinoma	^292^, ^293^, ^294^, ^295^
	Non‐small cell lung carcinoma	^217^, ^296^, ^297^
MCM7	Nonfunctioning pituitary adenomas	^298^
	Gastric cancer	^299^, ^300^, ^301^
	Hepatocellular carcinoma	^302^, ^303^, ^304^
	Meningiomas	^305^, ^306^, ^307^
	Prostate cancer	^308^
	Oral squamous cell carcinoma	^309^, ^310^
MCM8	Gastric cancer	^311^
	Cervical cancer	^313^
	Bladder cancer	^312^
MCM9	Colorectal cancer	^314^
MCM10	Ovarian cancer	^315^
	Breast cancer	^316^, ^317^, ^318^
	Hepatocellular carcinoma	^319^, ^320^
	Urothelial carcinoma	^320^, ^321^

Compared with the common‐used proliferation biomarker, MCM proteins are more sensitive and particular than PCNA and Ki‐67,^323^ accurately reflecting cell proliferation status and predicting prognostic tumor patient's survival rate.

### 6.3 MCMs as therapeutic target

Since increased DDR preventing cancer cells from effective therapy, MCM2‐7 proteins as intermediates are involved in intricate progression, which may serve as a pivotal role in influencing therapy response.

Conventional chemotherapy is one of the staple cancer treatment strategies, with emerging resistance causing the limited anticancer effects.^324^ Previous reviews highlighted that chemotherapy uniting with MCMs knockout approach inhibits tumor cell proliferation.^325^ Recently, combination chemotherapies have been widely used in cancer treatment, especially with the knockdown of MCM proteins. Carboplatin‐based chemotherapy is for the initial treatment of ovarian cancer, while carboplatin resistance results in treatment failure.^326^ Deng et al.^327^ found that knockdown of MCM2 could enhance the carboplatin sensitivity of A2780 cells and increase cells’ UV sensitivity, which may owe to accumulation of damaged DNA and activation of the p53‐dependent apoptotic response. Oxaliplatin or etoposide‐mediated chemotherapy combined with knockdown of MCM7 could reduce the proliferation of colorectal carcinoma cells and induce tumor apoptosis in vitro.^328^ In pancreatic ductal adenocarcinoma cells, reduction of MCM4 or MCM7 clearly exhibits more sensitive to gemcitabine and 5‐FU exposure, which may be caused by MCM suppression‐induced RS.^328^ Classic hypercholesterolemia curative simvastatin was demonstrated that reduces the expression of MCM7.^329^ Thus, it implied that simvastatin combining with chemotherapeutic drugs may be the putative cancer therapy for some chemo‐resistance cancer treatment. Liang et al.^330^ demonstrated that simvastatin combines with tamoxifen impaired breast cancer cell proliferation and resulted in apoptosis in vivo and in vitro. Knockout of MCM8 or MCM9 selectively increases cisplatin sensitivity in specific cancer cells such as HCT116 and HeLa cells.^331^, ^332^ However, siRNA‐mediated MCM8 silencing could not alter the cisplatin sensitivity of normal HFF2/T fibroblasts, indicating MCM8 may act as a molecular target just in cancer cells. Consistent with cisplatin, silencing MCM8 and MCM9 selectively hypersensitizes cancer cells to Olaparib, which may rely on MCM8‐9's role in resolving RS.^333^, ^334^

Radiotherapy is an alternative cancer therapy known to induce cancer cells’ DNA damage and autophagy to reach the treatment goals, while acquired radio‐resistance disturbs the effectiveness.^335^ Next‐generation sequencing of mRNA (RNA‐seq) results revealed that MCM7 is significantly upregulated in radio‐resistant PC‐3 cells after 2Gy IR treatment. Diminishing expression of MCM7 might increase radiotherapy response in prostate cancer.^336^ Consistent with this notion that upregulated expression of MCM2 is involved in the radio‐resistant cervical cancer cell, indicating that MCM2 is one potential regulatory factor in increasing radio‐sensitivity in cancer treatment.^337^ As mentioned above, MCM3 is one prognosis marker for HCC with high expression in vitro and in vivo. Using MTT and TUNEL methods, low MCM3 expression HCC cell line performs low growth, whereas high MCM3 expression induces lower apoptosis under radiotherapy. In addition, overexpression of MCM3 further promotes HCC cell radio‐resistance, revealing MCM3 prevents HCC radiotherapy efficiency via activating NF‐κB pathway.^259^

Specific small molecule inhibition for MCM proteins is an additional invaluable approach for various cancer treatments.^338^ There are three typical MCMs‐based small molecule inhibitors with potential chemotherapeutic effects: (a) Enzyme inhibitors such as DNA helicase‐targeting small molecule inhibitors.^339^ Ciprofloxacin targets MCM2‐7 complex to block the helicase activity, further inhibiting cell proliferation.^340^, ^341^ (b) The inhibitors prevent interaction among MCM subunits.^112^, ^342^, ^343^ (c) The inhibitors regulate the expression of MCM proteins.^344^ Widdrol could downregulate the expression of MCM proteins to inhibit cancer cell proliferation in G1 phase.^345^, ^346^ In addition, trichostatin A targets MCM2 to inhibit its expression.^347^, ^348^ Recent studies revealed that Breviscapine downregulates the expression of MCM7 and impairs tumor progression in prostate cancer via activating DNA damage‐induced apoptosis (Table 4).^349^

**TABLE 4:** MCM proteins as therapeutic target in different therapeutic scheme

Therapy	Cancer type	Target protein	Therapeutic scheme	Reference
Chemotherapy	Ovarian cancer	MCM2	Carboplatin	^326^, ^327^
	Colorectal carcinoma	MCM7	Oxaliplatin	^328^
	Pancreatic ductal adenocarcinoma	MCM4, MCM7	Gemcitabine/5‐FU	^328^
	Breast cancer	MCM7	Tamoxifen/Simvastatin	^329^, ^331^, ^332^
	Ovarian cancer	MCM8, MCM9	Olaparib	^333^, ^334^
Radiotherapy	Prostate cancer	MCM7	IR	^336^
	Cervical cancer	MCM2	IR	^337^
	Hepatocellular carcinoma	MCM3	IR	^259^
Small molecule inhibitor		MCM2‐7	Ciprofloxacin	^339^
		MCM2‐7	Widdrol	^345^, ^356^
		MCM2	trichostatin A	^347^, ^348^
		MCM7	Breviscapine	^349^

### 6.4 GINS and Cdc45 as prognostic markers and the target for therapy

Except for MCMs, concurrent overexpression of GINS and Cdc45 are also observed in various cancers. Since Psf1 promoter activity is related with 17β‐estradiol (E2)‐based estrogen receptor pathway, aberrant expression of Psf1 might be a signal in breast cancer. Nakahara et al.^350^ revealed that expression of Psf1 is remarkably increased in breast cancer both in vivo and in vitro, whereas siRNA‐mediated depletion of Psf1 inhibits DNA replication and cell growth. This evidence provides that Psf1 could be deemed as a novel breast cancer biomarker and therapeutic benefit for breast cancers with overexpression of Psf1.^350^ Moreover, uprelated expression of Psf1 is also detected in non‐small cell lung cancer, which implies Psf1 as a prognostic biomarker and potential target for lung cancer therapy.^351^, ^352^ Using the tumor cell xenograft model, Nakahama et al. found higher expression of Psf1 is correlative with higher proliferative ability and metastatic capability, implicating Psf1 in tumorigenesis and its conceivable role as a therapeutic target. Furthermore, anomalous expression of Psf1 also exists in prostate cancer and HCC,^353^, ^354^ significantly correlated with tumor grade and clinical stage.

Using cDNA microarray analysis, Psf2 is frequently upregulated in cholangiocarcinoma, while knockdown of Psf2 drastically reduces cell proliferation and inhibits cell growth.^355^ Furthermore, obvious upregulation of Psf2 is detectible in cervical cancer cell, whereas downregulation of Psf2 inhibits cell multiplication and tumorigenic ability.^356^ Combined with the observations, Psf2 might be a novel and valuable prognostic biomarker for ovarian cancer and leukemia,^357^, ^358^ subsequently an underlying molecular target for cancer diagnosis and treatment. In triple negative breast cancer (TNBC) cell lines, similar with Psf1, the expression of Psf2 is also enriched correlated with the advanced stages of tumor. Intriguingly, silencing of Psf2 decreases the expression of matrix metallopeptidase 9, which is necessary for tumor invasion, hence suppress tumor cell migration and invasion.^359^

Aberrant expression of Psf3 in colon carcinoma cell line associates with tumor cell proliferation and tumor progression, simultaneously, the similar results have also been found in non‐small cell lung cancer, lung adenocarcinoma, and colorectal cancer patients.^360^, ^361^, ^362^, ^363^ Finally, abundant Sld5 expression is closely related to bladder cancer and gastric cancer, since defect of Sld5 causes severe tumor cell proliferation disorder and inhibits cell growth.^364^, ^365^

Upregulated expression of Cdc45 is also a predictor for multiple cancers, including breast cancer, leukemia, lung cancer and osteosarcoma.^366^, ^367^ Using the Cancer Genome Atlas, prominent overexpression of Cdc45 is discovered in papillary thyroid cancer tissue, which impacts tumor sizes and cancer stages.^368^ In tongue squamous cell carcinomas (SCC), higher expression of Cdc45 with severe lymph node status is observed in malignant tumor than mild precancerous epithelial dysplasia, which implies its role in distinguish precancerous dysplasia from SCC.^369^ Myc is one crucial factor in regulating cell growth and tumorigenesis, overexpression of Myc enhances the Cdc45 DNA binding ability and replication fork stalling, which indicates Myc‐induced RS collaborates with Cdc45 in tumor development.^370^, ^371^

In summary, excessive expression of CMG is generally related with tumor size and malignancy but to varying extents, depending on the type of tumor. Thus, except for MCMs, GINS and Cdc45 could also serve as diagnostic and prognostic biomarkers for multiple tumors, which also as a druggable target to treat cancers (Table 5).

**TABLE 5:** GINS, Cdc45, and PCNA as prognostic biomarker

Proteins	Cancer type	Reference
Psf1	Breast cancer	^350^
	Non‐small cell lung cancer	^351^, ^352^
	Prostate cancer	^353^
	Hepatocellular carcinoma	^354^
Psf2	Cholangiocarcinoma	^355^
	Cervical cancer	^356^
	Ovarian cancer	^357^
	Leukemia	^358^
	Breast cancer	^359^
Psf3	Non‐small cell lung cancer	^360^, ^361^
	Lung adenocarcinoma	^363^
	Colorectal cancer	^362^
Sld5	Bladder cancer	^364^
	Gastric cancer	^365^
Cdc45	Breast cancer	^366^
	Leukemia	^366^
	Lung cancer	^366^, ^367^
	Osteosarcoma	^366^
	Thyroid cancer	^368^
	Tongue squamous carcinoma	^369^
PCNA	Lung cancer	^374^
	Prostate cancer	^375^
	Breast cancer	^376^
	Colorectal cancer	^377^
	Cervical cancer	^377^, ^378^
	Esophageal squamous carcinoma	^378^
	Hepatocellular carcinoma	^379^

Cancer immunotherapy is a novel biological treatment for malignant tumors, which could be collaborated with traditional radiotherapy and chemotherapy to improve the curative rate. Cancer immunotherapy elicits the immune system by identifying specifically expressed molecules on the tumor surface, thereby eliminating the cancer cells.^372^ Cytotoxic T lymphocytes (CTLs) are specific T cells with potent nocuity to cancer cells, which recognizing cancer‐specific antigenic peptides human leukocyte antigen (HLA). Though mass spectrometric analyses and bioinformatic analysis, Yoshida et al.^373^ isolates a Psf1‐derived peptide presented by HLA. Moreover, they found no other cancer vaccine target proteins except for Psf1. Detailed peptide is verified using the mouse model, suggesting Psf1_79–87_ peptide induces CTL response in vitro and in vivo, which provides a novel cancer immunotherapy for targeting cancer stem cells. Previously, the similar approach was utilized in Cdc45, demonstrating that strongly immunogenic Cdc45‐derived peptides stimulated CTLs to be reactive to lung cancer cells.^373^

### 6.5 PCNA as prognostic markers and the target for therapy

Due to its role in cell proliferation, PCNA is deemed as the tumor marker for diagnosis and patient prognosis. Overexpression of PCNA is observed in lung cancer in vitro and in vivo, while silencing of PCNA reduces cell invasion ability and 95D cells proliferations.^374^ Identical results also occur in prostate carcinoma and breast cancer that PCNA connects with pathological stage and cellular grade, suggesting PCNA might be a crucial prognostic indicator of malignant tumors.^375^, ^376^ Furthermore, upregulated PCNA is associated with various digestive system tumors including colorectal carcinoma, ESCC, and HCC (Table 5).^377^, ^378^, ^379^

PCNA is a critical factor in DNA replication and DNA damage repair. Hence, PCNA is a target for designing antiproliferation and anticancer drugs. Since multiple PTMs of PCNA interrupt its chromatin binding ability, developing therapeutics of modified PCNA will be conducive to target cancer cells. Previous research found that Y211 phosphorylation of PCNA is a crucial event to preserve the PCNA stability, promoting DNA damage repair and DNA synthesis. Therefore, mutant of Y211 phosphorylation inhibits tumor cells proliferation including prostate cancer and breast cancer.^380^, ^381^ In addition, specific small molecular inhibitors targeting PTMs of PCNA are also identified to disrupt cell proliferation and enhance chemosensitivity or radiosensitivity. RAD6 selective small molecular inhibitor SMI#9 leads to PCNA mono‐Ub defect and mitochondrial function reduction, suggesting RAD6 inhibitor serves as a promising strategy for TNBC treatment.^382^

Although PTMs of PCNA have several implications in carcinogenesis; however, the detailed antitumor mechanism needs further investigation. Moreover, novel inhibitors or strategies inhibiting tumor development or proliferation via targeting PTMs of PCNA require identification.

## 7 CONCLUSION AND PERSPECTIVES

Numerous proteins perform DNA replication to maintain genetic information transmission from the parental generation to the next generation. In line with this, DNA replication factors are strongly associated with DDR to ensure genome integrity. Abnormal expression of DNA replication proteins results in genomic instability and performs a surprising diversity of symptoms. In this review, we outline the DNA replication mechanism and review the distinct roles of replication factors in DNA replication, DDR, and tumorigenesis.

Increasing publications revealed abundant replication factors are involved in DDR upon RS. Dynamic status of these proteins is regulated by some kinases, such as ATR and ATM, while multiple PTMs also modulate their functions and architecture. However, PTM‐mediated structure alternation may also contribute to DNA topological change to assist chromosomal rearrangement.^383^ As we summarized, PTMs of MCMs and PCNA impact critical integration of DNA replication, DDR, and even cancer therapy. Nevertheless, the temporospatial modification crosstalk and definite modification sites still need deep investigation. It will be fascinating to clarify the crosstalk among distinct PTMs and map a trenchant signal network of DNA replication licensing and DNA damage repair.

Nevertheless, loss and mutation of the DNA replication factors is also the source of various disorders. In mammals, loss of function of polη leads to a hereditary disease xeroderma pigmentosum variant (XPV), which is characterized by a high frequency of skin cancer.^384^ In addition, mutations in ORC complex cause the developmental disorder Meier‐Gorlin syndrome and Wolf‐Hirschhorn syndrome.^385^ These atypical diseases with defects in DNA replication are still a tough job to explore in the future.

Dysregulation of DNA replication in cancer cells causes carcinogenesis with aberrant expression of replication proteins. Thus, such replication factors are closely related to tumorigenesis and development, acting as diagnostic and prognostic biomarkers among multiple tumors. Based on numerous biological research, overexpression of CMG and PCNA disturbs routine cell proliferation and regular DNA damage repair, promoting tumor development. Therefore, target therapy may be a potential approach to cancer treatment. In this review, we also arranged the current understanding of combination anticancer strategies with knockdown of replication factors. With the rapid evolution of pharmaceutics, small molecule inhibitions targeting these proteins are also designed for clinical application. Considering the PTMs of these proteins are also involved in DNA replication and their activity, PTMs of replication proteins may be a putative field to design associate small molecule inhibitors.

## AUTHOR CONTRIBUTIONS

Hao‐yun Song was responsible for the manuscript. Hao‐yun Song designed the project in collaboration with De‐gui Wang and Rong Shen. Hao‐yun Song wrote the manuscript. Ya‐nan Guo, Hamid Mahasin, Rong Shen, and De‐gui Wang revised the manuscript. All authors have read and approved the final manuscript.

## CONFLICT OF INTEREST

The authors declare that they have no known competing financial interests or personal relationships that could have appeared to influence the work reported in this paper.

## ETHICS STATEMENT

Not applicable.

## Data Availability

Not applicable.
